# Hyperlactatemia during cardiopulmonary bypass: determinants and impact on postoperative outcome

**DOI:** 10.1186/cc5113

**Published:** 2006-11-29

**Authors:** Marco Ranucci, Barbara De Toffol, Giuseppe Isgrò, Federica Romitti, Daniela Conti, Maira Vicentini

**Affiliations:** 1 Department of Cardiovascular Anesthesia and Intensive Care, IRCCS Policlinico S. Donato, Via Morandi 30, 20097 San Donato Milanese, Milan, Italy

## Abstract

**Introduction:**

Hyperlactatemia during cardiopulmonary bypass is relatively frequent and is associated with an increased postoperative morbidity. The aim of this study was to determine which perfusion-related factors may be responsible for hyperlactatemia, with specific respect to hemodilution and oxygen delivery, and to verify the clinical impact of hyperlactatemia during cardiopulmonary bypass in terms of postoperative morbidity and mortality rate.

**Methods:**

Five hundred consecutive patients undergoing cardiac surgery with cardiopulmonary bypass were admitted to this prospective observational study. During cardiopulmonary bypass, serial arterial blood gas analyses with blood lactate and glucose determinations were obtained. Hyperlactatemia was defined as a peak arterial blood lactate concentration exceeding 3 mmol/l. Pre- and intraoperative factors were tested for independent association with the peak arterial lactate concentration and hyperlactatemia. The postoperative outcome of patients with or without hyperlactatemia was compared.

**Results:**

Factors independently associated with hyperlactatemia were the preoperative serum creatinine value, the presence of active endocarditis, the cardiopulmonary bypass duration, the lowest oxygen delivery during cardiopulmonary bypass, and the peak blood glucose level. Once corrected for other explanatory variables, hyperlactatemia during cardiopulmonary bypass remained significantly associated with an increased morbidity, related mainly to a postoperative low cardiac output syndrome, but not to mortality.

**Conclusion:**

Hyperlactatemia during cardiopulmonary bypass appears to be related mainly to a condition of insufficient oxygen delivery (type A hyperlactatemia). During cardiopulmonary bypass, a careful coupling of pump flow and arterial oxygen content therefore seems mandatory to guarantee a sufficient oxygen supply to the peripheral tissues.

## Introduction

Hyperlactatemia (HL) is a well-recognized marker of circulatory failure, and its severity has been associated with mortality in different clinical conditions [1,2]. After cardiac surgery, HL is relatively common [3,4] and is associated with morbidity and mortality [4]. During cardiac surgery with cardiopulmonary bypass (CPB) in adult patients, HL is detectable at a considerable (10% to 20%) rate [5,6] and is associated with postoperative morbidity and mortality [5]. At present, the nature of HL during and after cardiac operations is not totally clear, but the majority of authors [4,7-9] tend to attribute this finding to a tissue hypoxia (type A HL) even if type B HL (without tissue hypoxia) has been advocated in some cases [10]. The main factors leading to a possible organ dysoxia during CPB are the hemodilution degree [11,12] and a low peripheral oxygen delivery (Do_2_) [13]. Both have been associated with postoperative morbidity and mortality. Hence, there is a consistent body of information suggesting that during CPB an unrecognized pattern of critically decreased peripheral oxygen supply may occur and that, as a result of this condition of circulatory failure, lactate production appears. As a matter of fact, the concept of critical Do_2 _is based on the assumption that when a patient is perfused below the critical value, the oxygen consumption (Vo_2_) becomes dependent on the Do_2 _[14-16] and energy production is partially supplied by anaerobic glycolysis. As a result, lactate production increases and HL takes its course [17,18].

Despite this apparently reasonable assumption, no scientific evidence of an association between HL and oxygen supply during CPB is available. Even the association between HL during CPB and postoperative morbidity/mortality is far from being well defined, the only report being based on a retrospective study [5]. The present study was designed with two endpoints: (a) to define the factors associated with HL during CPB, specifically with respect to perfusion-related factors during CPB, and (b) to verify the clinical impact of HL during CPB in terms of postoperative morbidity and mortality.

## Materials and methods

### Study design

This was a prospective observational study conducted at our institution from September 1 2005 to December 22 2005. The study design did not include any intervention, and data collection was based on the local database and routine measurements performed during the operation. Therefore, the local ethical committee waived the need for approval. All of the patients gave written consent to the scientific treatment of their data.

### Patient population

Five hundred consecutive adult patients (age > 18 years) undergoing cardiac surgery operations were admitted to this study. No operation-based selection was applied (excluding cardiac transplantation that is not performed at our institution). The only exclusion criterion was the presence of an abnormal (> 2 mmol/l) plasma lactate value before entering CPB. This condition, generally associated with emergency procedure, unstable preoperative hemodynamics, and pre- or intraoperative need for inotropic support or intra-aortic balloon pump, was detected in 30 patients, who were therefore excluded from the subsequent analyses. The remaining 470 patients were analyzed according to the purposes of the study.

### Anesthesia, surgery, and CPB management

Premedication included atropine sulphate (0.5 mg), prometazine (50 mg), and fentanyl (50 to 100 μg according to the patient's weight) intramuscularly administered one hour before the induction of anesthesia. Anesthesia was induced with an intravenous infusion of remifentanil (starting dose 0.5 μg/kg per minute) and a midazolam bolus of 0.2 mg/kg. Cisatracurium besylate (0.2 mg/kg) was subsequently administered to allow tracheal intubation. Subsequently, the anesthesia was maintained with a continuous infusion of remifentanil (dose ranging from 0.05 to 1 μg/kg per minute, titrated on the basis of the hemodynamic response) and midazolam (0.1 mg/kg per hour).

CPB was established via a standard median sternotomy, aortic root cannulation, and single or double atrial cannulation for venous return. Lowest core body temperature during CPB varied from 27°C to 37°C as requested by the surgeon. Antegrade intermittent cold crystalloid or cold blood cardioplegia was used according to the surgeon's preference. The circuit was primed with 700 ml of a gelatin solution (Medacta Italia, Milan, Italy) and 200 ml of trihydroxymethylaminomethane solution. Roller (Stöckert, now part of Sorin Group Deutschland GmbH, München, Germany) or centrifugal (Medtronic, Inc., Minneapolis, MN, USA) pumps were used according to availability; a biocompatible treatment (phosphorylcholine coating) and a closed circuit with separation of the blood suctions were used in 20% of the patients. The oxygenator was a hollow fiber D 905 Avant (Dideco, now part of Sorin Group Italia S.r.l. Mirandola, Italy). The pump flow was targeted between 2.0 and 2.4 l/minute per m^2 ^and the target mean arterial pressure was settled at 60 mm Hg. The gas flow was initially settled at 50% oxygen/air ratio and a 1:2 flow ratio with the pump flow indexed and was subsequently arranged in order to maintain an arterial oxygen tension greater than 150 mm Hg and an arterial carbon dioxide tension between 33 and 38 mm Hg.

Anticoagulation was established with an initial dose of 300 IU per kilogram of body weight of porcine intestinal heparin injected into a central venous line ten minutes before the initiation of CPB and with a target activated clotting time of 480 seconds; patients receiving closed and biocompatible circuits received a reduced dose of heparin with a target activated clotting time settled at 300 seconds. At the end of CPB, heparin was reversed by protamine chloride at a 1:1 ratio of the loading dose, regardless of the total heparin dosage.

### Data collection and definitions

The following preoperative data were collected and analyzed: demographics (age [years], gender, weight [kg], and height [cm]), preoperative cardiovascular profile (ejection fraction, New York Heart Association functional class, recent [30 days] myocardial infarction, unstable angina, congestive heart failure, previous vascular surgery, previous cardiac surgery, cardiogenic shock, use of intra-aortic balloon pump, and active endocarditis), presence of comorbidities (chronic renal failure, diabetes on medication, chronic obstructive pulmonary disease, and cerebrovascular accident), and laboratory assays (serum creatinine value [mg/dl] and hematocrit [HCT] [percentage]).

Operative data comprised type of operation (isolated coronary artery bypass graft, isolated valve procedure, and combined operation), CPB duration (minutes), lowest temperature (°C), and lowest pump flow indexed reached on CPB. At the onset of CPB and every 20 minutes, an arterial blood gas analysis, including blood glucose (mg/dl) and lactate (mmol/l) determination, was obtained. Blood gas analyses were performed using a Nova Stat Profile blood gas analyzer (Nova Biomedical Corporation, Waltham, MA, USA). On the basis of the arterial blood data, we assessed the lowest HCT (percentage) on CPB, the lowest Do_2 _(ml/minute per m^2^) on CPB (calculated according to standard equations on the basis of arterial hemoglobin concentration and saturation and on pump flow indexed), the peak blood glucose, and the peak lactate concentration.

Outcome variables included time on mechanical ventilation (MV) (hours), intensive care unit (ICU) stay (days), postoperative hospital stay (days), peak postoperative serum creatinine level (mg/dl), surgical revision rate, perioperative myocardial infarction rate (new Q waves plus enzymatic criteria), low cardiac output syndrome, atrial fibrillation rate (not pre-existing), presence of ventricular arrhythmias, acute renal failure (requiring renal replacement therapy), stroke, severe pulmonary dysfunction, cardiac arrest, sepsis, composite morbidity index (one of the following major complications: surgical reoperation, need for intra-aortic balloon pump, stroke, acute renal failure, or sepsis), and hospital mortality rate. In accordance with previous studies [4,6], HL was defined as a peak blood lactate value greater than 3 mmol/l.

### Statistical analysis

All data are expressed as mean ± standard error of the mean or as absolute numbers and percentage when appropriate. A *p *value less than 0.05 was considered significant for all of the following statistical tests. The statistical analysis was performed using SPSS 11.0 software (SPSS Inc., Chicago, IL, USA).

Univariate association with peak blood lactate was tested with a correlation matrix. Factors significantly (*p *< 0.05) associated with the peak blood lactate at this preliminary step were entered into a stepwise forward multivariable linear regression analysis, with adequate corrections to avoid multicollinearity within the model. The multivariable approach was applied to assess the independent association between the variables tested and the peak blood lactate. Subsequently, the population was explored in terms of HL (> 3 mmol/l) incidence.

A graphical analysis of the relationship between intraoperative variables and peak blood lactate value was performed using a non-linear regression analysis based on the technique of 'rolling decile' subgroups [11,19]. This technique is based on the following steps: (a) the patient population is ordered according to the independent variable tested (lowest Do_2 _on CPB, peak blood glucose, and CPB duration), (b) the population is divided into deciles and subsequently into 37 rolling deciles (having 75% overlapping ranges), (c) the mean value of the independent variables and the corresponding mean value of the peak blood lactates are calculated, and (d) the 37 points are plotted separately for the three independent variables. The rationale for this approach is to create a clear graphical relationship avoiding the difficult and confounding use of a standard plot of the original 470 experimental points. The patient population was arranged in order of increasing peak blood glucose levels, lowest Do_2_, and CPB duration, and a total of 37 subgroups (75% overlapping ranges) were analyzed with respect to the HL incidence. The same three intraoperative variables were tested for predictivity of HL by using a receiver operating characteristic (ROC) analysis. Postoperative outcome was firstly analyzed in the population with or without HL during CPB by using a univariate approach (Student's *t *test for unpaired data or relative risk analysis) and was subsequently corrected for other covariates in a multivariable linear or logistic regression analysis.

## Results

Preoperative profile and operative data of the patient population are reported in Table 1.

Twelve pre- and intraoperative factors were found to be significantly associated with the peak blood lactate level during CPB at the univariate analysis (Table 2). Age, ejection fraction, isolated coronary operation, lowest pump flow, lowest temperature, HCT, and Do_2 _during CPB were negatively correlated to the peak blood lactate value during CPB. Presence of active endocarditis and congestive heart failure, preoperative serum creatinine level, CPB duration, and peak blood glucose during CPB were positively correlated to the peak blood lactate value during CPB.

**Figure 1:**
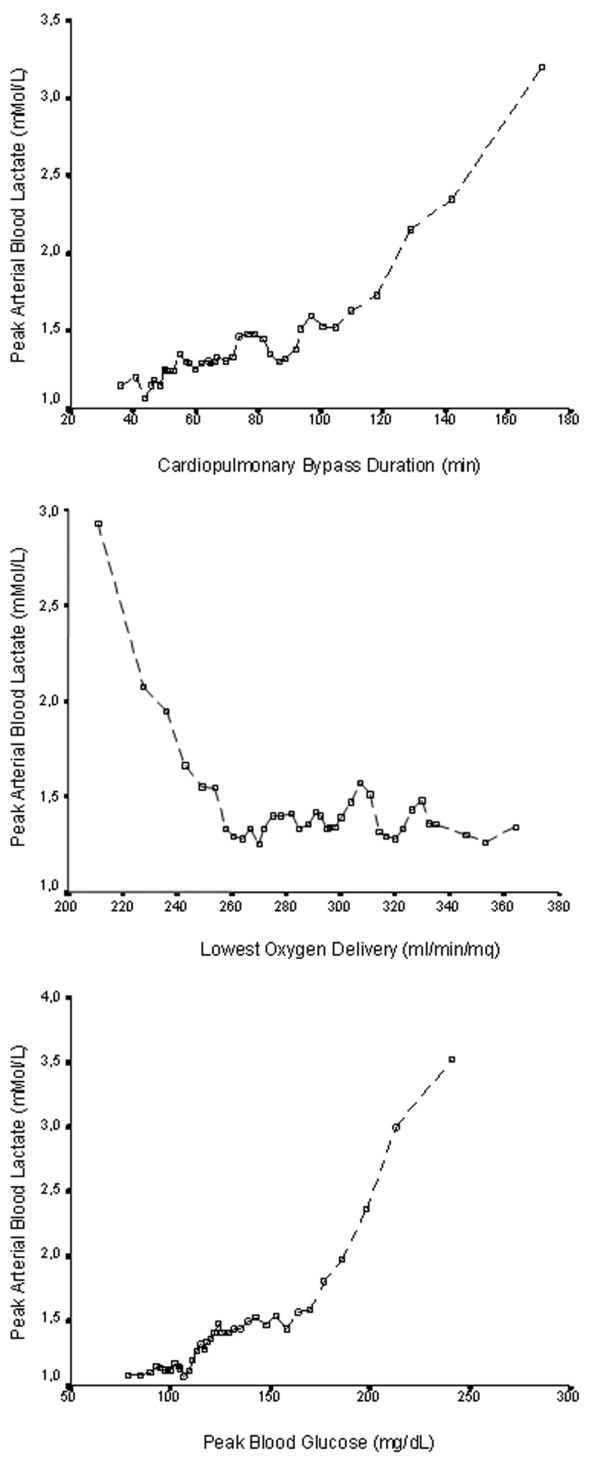
Peak arterial blood lactate value during cardiopulmonary bypass according to the cardiopulmonary bypass duration, the lowest oxygen delivery, and the peak blood glucose. Data are shown as rolling deciles (75% overlapping). Symbols (open boxes) represent the mean value recorded for each decile.

Some of these factors demonstrated a significant intercorrelation (ejection fraction with congestive heart failure; lowest pump flow and lowest HCT with the lowest Do_2 _during CPB). To avoid multicollinearity, the most significant factors (ejection fraction and Do_2 _during CPB) were included in the multivariable analysis, whereas the others were discharged. In the resulting multivariable stepwise forward linear regression analysis (Table 2), five factors remained independently associated with the peak blood lactate value (preoperative serum creatinine level, presence of active endocarditis, CPB duration, lowest Do_2 _during CPB, and peak blood glucose level during CPB). The last three factors were explored using a rolling decile graphical analysis (Figure 1). When analyzed with best-fit equations, quadratic non-linear regressions demonstrated the best fit.

The same intraoperative factors were tested for predictivity of HL (Table 3) with an ROC analysis. The area under the curve was significant for all three factors. However, no cutoff value could be detected for the lowest Do_2 _during CPB; conversely, cutoff values of 96 minutes for CPB duration (sensitivity 74%, specificity 80%) and of 160 mg/dl for peak blood glucose on CPB (sensitivity 84%, specificity 83%) were found.

HL was detected in 27 (5.7%) patients, and hyperglycemia (> 160 mg/dl) in 92 (19.6%). The patient population was analyzed according to the presence of HL, hyperglycemia, or both, with respect to the peak blood lactates and to the lowest Do_2 _on CPB (Table 4). Patients without HL or hyperglycemia had significantly lower values of peak blood lactate than the other three groups; patients with both HL and hyperglycemia had significantly higher peak blood lactate values than patients with only HL or hyperglycemia. Only the patients with associated HL and hyperglycemia had significantly lower values of Do_2 _on CPB.

Outcome variables associated with the presence of HL during CPB were MV time and need for prolonged (> 48 hours) MV, ICU stay and need for prolonged (> 7 days) ICU stay, postoperative peak serum creatinine level, need for surgical revision, need for intra-aortic balloon pump, incidence of atrial fibrillation, severe lung dysfunction, sepsis, composite morbidity index, and hospital mortality (Table 5). The univariate model was then corrected for the other covariates determining the peak blood lactate value (preoperative serum creatinine value, presence of active endocarditis, and CPB duration). After correction in a multivariable linear or logistic regression analysis, the outcome variables significantly associated with HL during CPB were ICU stay, need for intra-aortic balloon pump, and the composite morbidity index. Patients with HL during CPB had a significantly higher rate of prolonged MV time and ICU stay (Table 5). Patients with hyperglycemia not associated with HL were separately investigated for the outcome variables. No significant differences in terms of morbidity or mortality were detected in association with this isolated condition.

## Discussion

The main findings of our study are that HL during CPB (a) is more likely to occur in procedures requiring a prolonged CPB time, (b) appears to be independently associated with a low Do_2_, (c) is almost invariably associated with hyperglycemia, and (d) is a marker of a worse postoperative outcome in terms of morbidity, even if it is not significantly associated with an increased mortality rate.

The rate of patients demonstrating HL during CPB was relatively low (5.7%). However, in this study, we focused on HL progressively established during CPB and excluded 30 patients who entered CPB with a pre-existing HL. The overall incidence of HL was 11.4%, which is still lower than the one reported by previous studies [5].

Various preoperative factors or comorbidities may create the right environment for HL during CPB. Age, female gender, congestive heart failure, low left ventricular ejection fraction, hypertension, atherosclerosis, diabetes, preoperative hemoglobin value, redo or complex surgery, and emergency procedures were found to be risk factors for HL by Demers and coworkers [5], who reported an HL incidence of 18%. Some of these factors were confirmed in our study, and other new factors were identified; however, our study population had a significantly shorter CPB duration and a lower degree of hemodilution during CPB. Given that both these factors seem to favor the onset of HL, the lower HL rate in our population is reasonably explained.

The role of CPB duration in the determinationof HL during CPB has been highlighted by other authors [5]. In fact, the association between CPB duration and peak blood lactate level is not linear: we could identify a cutoff value of 96 minutes as predictive of HL during CPB.

On the basis of our data, the main rationale for explaining HL during CPB is a Do_2 _inadequate to guarantee the needed Vo_2 _of the patient. The association between the lowest Do_2 _during CPB and HL is maintained within a multivariable model, and the predictivity of the lowest Do_2 _is confirmed by the ROC analysis. We could not identify a specific cutoff value, but from the graphical relationship obtained using the rolling decile technique, the value of Do_2 _below which the peak blood lactate starts increasing is approximately 260 ml/minute per m^2^.

There are no previous studies addressing Do_2 _and lactate levels during CPB. However, Demers and coworkers [5] found that a low hemoglobin level during CPB is associated with HL, and it is reasonable to interpret this information within the context of a low Do_2 _during CPB.

The link between Do_2 _and HL definitely defines HL during CPB as type A HL. It appears reasonable that under certain circumstances (favored by some preoperative comorbidities) and in the presence of a prolonged CPB, the Do_2 _may decrease below a critical level, the Vo_2 _becomes dependent on the Do_2 _and starts decreasing, and lactic acidosis is established.

Interestingly, in a previous study [13], we could demonstrate that the incidence of acute renal failure after cardiac operations is significantly increased in patients perfused below the critical Do_2 _value of 272 ml/minute per m^2^, a figure that appears to be in agreement with the data of the present study. This information, together with the well-known association between severe hemodilution during CPB and bad outcomes [11,12], reinforces the interpretation that patients with HL during CPB are suffering from a sort of masked circulatory shock, which will exert its deleterious effects on different organs (mainly on renal function) during the early phases of the postoperative course.

The association between hyperglycemia and HL may be interpreted within this model of circulatory failure during CPB. In a model of cardiogenic shock after heart surgery, Chioléro and coworkers [20] could demonstrate that HL is due mainly to increased production rather than to impaired lactate use. HL was almost invariably accompanied by hyperglycemia due mainly to increased glucose production, which was probably due to the release of stress hormones and cytokines leading to insulin resistance [21]. The extra amount of glucose fails to enter the oxidative pathway and is degraded to lactate by the glycolytic pathway.

In our model, a possible interpretation is that a reduced Do_2 _due to insufficient pump flow, severe hemodilution, or both creates a condition similar to a cardiogenic shock, leading on one side to a direct lactate formation by the dysoxic organs and on the other to a catecholamine release, insulin resistance, hyperglycemia, and lactate formation (with subsequent liver uptake and reconversion to glucose by the Cori cycle).

The link between HL and hyperglycemia through the mechanism explained above was confirmed by the same group of researchers in 2005 [22] in an elegant study dealing with cardiogenic or septic shock. The role of adrenergic agonists in this setting is well defined: in cardiogenic shock, they are both endogenous or administered for cardiovascular therapy; in our model, they are endogenous in the majority of the patients. None received epinephrine during CPB, and few received norepinephrine; however, unlike epinephrine, norepinephrine usually does not increase glucose production or induce an increase in plasma lactate concentration [23].

The two mechanisms leading to HL in various clinical conditions are therefore (a) anaerobic metabolism due to a poor Do_2 _and (b) excess lactate production due to glucose failing to enter the oxidative pathway and being degraded to lactate by the glycolytic pathway. These mechanisms, if independently considered, lead to different acid-base balance conditions, the former being accompanied by metabolic acidosis and the latter not necessarily so. However, in the clinical conditions of this observational study, the acid-base balance is constantly maintained at a normal pH value by bicarbonate corrections applied by the perfusionist whenever the base excess starts decreasing. Therefore, we are unable to identify differences in pH related to different values of peak blood lactates. However, the evidence that only four patients demonstrated HL without hyperglycemia and that only patients with an HL-hyperglycemia syndrome had a significantly lower value of Do_2 _seems to confirm that, in our specific clinical environment, HL and hyperglycemia are linked by the causative factor of a poor Do_2_, leading on one side to lactate production through the anaerobic pathway and on the other to a vicious cycle of lactate production due to the poor ability to use glucose through the aerobic pathway.

Whenever the Do_2 _decreases, compensatory mechanisms are usually triggered to maintain the Vo_2 _through a higher oxygen extraction. Consequently, the mixed venous oxygen saturation (Svo_2_) decreases. The measurement of the Svo_2 _is possible during CPB, but very rarely is it routinely performed using on-line measurement devices in adult patients. Mixed venous blood gas analyses were available in our experimental setting but not at any arterial blood gas analysis time point. Therefore, in this study, we cannot address the association between Svo_2 _and blood lactates. However, in a previous study, we could demonstrate that under CPB conditions the correlation between the two variables was very poor [6].

In our series, HL during CPB leads to an increased morbidity that, after correction for other explanatory variables, appears to be related mainly to a low cardiac output state. This increased morbidity leads in turn to prolonged MV and ICU stay. Conversely, mortality is not significantly associated with HL.

Only one article addresses the association between HL during CPB and postoperative outcome [5]. In that work, HL was significantly associated with a number of morbid events and with mortality. However, the above data are not corrected for the other explanatory variables. When included into a multivariable model, general morbidity and mortality remained significantly associated with HL during CPB; unfortunately, the authors failed to indicate the odds ratios for both of the multivariable logistic regressions applied, making a comparison between their results and our results impossible. Our data suggest that HL is associated with morbidity but not with mortality; given that HL is more frequent in the presence of comorbidities and/or prolonged CPB time, the inclusion of these covariates into the predictive models reduces, but does not abolish, the role of HL during CPB in deteriorating the postoperative outcome in cardiac surgery. Of course, we cannot exclude that in a larger cohort of patients, HL during CPB may be confirmed as an independent risk factor for mortality too. Hyperglycemia not accompanied by HL was not a morbidity/mortality risk factor in our model.

As a final remark, we must consider that a CPB model of HL and Do_2 _offers some experimental advantages. Both the hemoglobin content and the pump flow are under the control of the operator and may be modulated by intervention. This was not the case with the present observational study, but this model could be used for future interventional studies. However, even if the study was conducted following the generally accepted standards of CPB management, some very low values of Do_2 _were observed (3% of the patients had a lowest Do_2 _< 200 ml/minute per m^2^) and these were related mainly to a pronounced hemodilution.

## Conclusion

HL during CPB is due mainly to a Do_2 _inadequate to fulfill the metabolic needs of the patient, and this critical value is approximately 260 ml/minute per m^2^. This 'circulatory shock' condition is associated with a reactive hyperglycemia that is probably due to insulin resistance triggered by a catecholamine release. The above condition plays a significant role in deteriorating the postoperative outcome. Therefore, every attempt should be applied to avoid HL during CPB, and the critical Do_2 _value of 260 to 270 ml/minute per m^2 ^should be considered whenever setting the pump flow and the maximum acceptable hemodilution degree.

## Key messages

• Non-pre-existing HL during CPB for cardiac operations in adults occurs in approximately 6% of the patients.

• It is favored by the preoperative risk profile (high serum creatinine values and active endocarditis) and by prolonged (> 96 minutes) CPB times.

• It is triggered by an inadequate Do_2 _and generally appears when the Do_2 _is less than 260 to 270 ml/minute per m^2^.

• It is associated with hyperglycemia.

• It is associated with an increased postoperative morbidity but not with mortality.

## Abbreviations

CPB = cardiopulmonary bypass; Do_2 _= oxygen delivery; HCT = hematocrit; HL = hyperlactatemia; ICU = intensive care unit; MV = mechanical ventilation; ROC = receiver operating characteristic; Svo_2 _= venous oxygen saturation; Vo_2 _= oxygen consumption.

## Competing interests

MR declares that he is the owner of a patent for a monitoring device during CPB. This device is not commercially available at present and has not been used for the purposes of the present study.

## Authors' contributions

MR participated in the study design, statistical analysis, and writing of the manuscript. BDT and MV participated in the data collection and references search. GI participated in the study design, statistical analysis, and manuscript preparation. DC and FR participated in the data collection, statistical analysis discussion, and manuscript preparation. All authors read and approved the final manuscript.

## Figures and Tables

**Table 1:** Preoperative profile and operative data

Variable	Number (percentage) or mean ± standard deviation
Age (years)	64.5 ± 14.2
Male gender	324 (69)
Body surface area (m^2^)	1.81 ± 0.18
Left ventricle ejection fraction	0.51 ± 0.12
Unstable angina	36 (7.7)
Previous vascular surgery	19 (4.1)
Previous cardiac surgery	39 (8.3)
Recent myocardial infarction	113 (24)
Congestive heart failure	30 (6.4)
Cardiogenic shock	5 (1.1)
Preoperative intra-aortic balloon pump	4 (0.8)
Active endocarditis	7 (1.5)
Dialytic treatment	4 (0.8)
Diabetes on medication	56 (12)
Chronic obstructive pulmonary disease	19 (4.1)
Previous cerebrovascular accident	33 (7)
Hematocrit (percentage)	39.8 ± 4.6
Serum creatinine value (mg/dl)	1.1 ± 0.8
Isolated coronary operation	211 (45)
Isolated valve operation	138 (29)
Combined operation	121 (26)
CPB duration (minutes)	81 ± 41
Lowest hematocrit on CPB (percentage)	27 ± 3.5
Lowest oxygen delivery on CPB (ml/minute per m^2^)	291 ± 44
Lowest temperature on CPB (°C)	31 ± 1.9
Peak blood lactate (mmol/l)	1.5 ± 1.3
Peak blood glucose (mg/dl)	133 ± 47

CPB, cardiopulmonary bypass.

**Table 2:** Univariate and multivariable analyses for pre- and intraoperative factors associated with peak blood lactate value

Univariate analysis (correlation matrix)		
Factor	Correlation coefficient	*p *value

Age (years)	-0.099	0.032
Ejection fraction	-0.181	0.001
Congestive heart failure	0.191	0.001
Preoperative serum creatinine value (mg/dl)	0.191	0.001
Active endocarditis	0.177	0.001
Isolated coronary operation	-0.094	0.041
CPB duration (minutes)	0.523	0.001
Lowest temperature (°C) on CPB	-0.312	0.001
Lowest hematocrit on CPB	-0.158	0.001
Lowest pump flow (l/minute per m^2^) on CPB	-0.271	0.001
Lowest Do_2 _(ml/minute per m^2^) on CPB	-0.276	0.001
Peak blood glucose (mg/dl) on CPB	0.517	0.001

Multivariable analysis (linear regression)		

Factor	B coefficient	*p *value

Constant	0.326	
Preoperative serum creatinine value (mg/dl)	0.123	0.032
Active endocarditis	0.177	0.001
CPB duration (minutes)	0.012	0.001
Lowest Do_2 _(ml/minute per m^2^) on CPB	-0.004	0.004
Peak blood glucose (mg/dl) on CPB	0.009	0.001

CPB, cardiopulmonary bypass; Do_2_, oxygen delivery.

**Table 3:** Receiver operating characteristic analysis for the three intraoperative predictors of hyperlactatemia

Factor	AUC	95% CI	*p *value	Cutoff value	Sensitivity	Specificity
Lowest Do_2 _on CPB	0.70	0.55–0.81	0.001	Undetectable	-	-
Peak blood glucose on CPB	0.91	0.86–0.96	0.001	160 mg/dl	84%	83%
CPB duration	0.80	0.68–0.89	0.001	96 minutes	74%	80%

AUC, area under the curve; CI, confidence interval; CPB, cardiopulmonary bypass; Do_2_, oxygen delivery.

**Table 4:** Subgroup analysis for peak blood lactates and lowest Do_2 _on CPB

	No HL or HG	HL alone	HG alone	HL and HG
	(*n *= 374)	(*n *= 4)	(*n *= 69)	(*n *= 23)
Peak blood lactate (mmol/l)	1.27 ± 0.46^a^	3.42 ± 0.85^b^	1.62 ± 0.61^c^	5.82 ± 3.34^d^
Lowest Do_2 _(ml/minute per m^2^)	294 ± 41	294 ± 53	287 ± 44	255 ± 62^e^

^a^*p *< 0.01 versus the other three groups; ^b^*p *< 0.01 versus groups HG alone and HL plus HG; ^c^*p *< 0.01 versus groups HL alone and HL plus HG; ^d^*p *< 0.01 versus groups HL alone and HG alone; ^e^*p *< 0.01 versus group no HL or HG and *p *= 0.014 versus group HG alone. CPB, cardiopulmonary bypass; Do_2_, oxygen delivery; HG, hyperglycemia; HL, hyperlactatemia.

**Table 5:** Hyperlactatemia during CPB and postoperative outcome

	Univariate analysis (Student's *t *test)			Corrected^a ^values
Outcome variable	No HL (*n *= 443)	HL (*n *= 27)	*p*	*P*

Peak serum creatinine (mg/dl)	1.3 ± 1.1	2.1 ± 1.4	0.001	0.45
MV time (hours)	22.6 ± 55	57.4 ± 68	0.015	0.41
ICU stay (days)	2.5 ± 3.4	5.3 ± 5.4	0.012	0.04

	Univariate analysis (RR)			Corrected^b ^values

Outcome variable	No HL (*n *= 443)	HL (*n *= 27)	RR (95% CI)	OR (95% CI)

Prolonged (> 7 days) ICU stay	18 (4.1%)	5 (18.5%)	5.3 (1.8–15.8)	4.2 (1.04–17)
Prolonged (> 48 hours) MV	21 (4.7%)	9 (33.3%)	10 (4–25)	4.9 (1.6–15)
Surgical revision	19 (4.3%)	5 (18.5%)	5.1 (1.7–14.8)	2.5 (0.6–9.5)
Intra-aortic balloon pump	2 (0.4%)	3 (11.1%)	27.5 (4.4–172)	23 (2.7–207)
Atrial fibrillation	75 (17%)	9 (33.3%)	2.4 (1.06–5.6)	1.6 (0.6–4.1)
Severe lung dysfunction	6 (1.3%)	2 (7.4%)	5.8 (1.1–30.3)	0.8 (0.1–8.5)
Sepsis	9 (2%)	3 (11.1%)	6 (1.5–23.7)	3.6 (0.6–21)
Composite morbidity index	36 (8.1%)	9 (33.3%)	5.6 (2.4–13.5)	2.9 (1.03–8.5)
Hospital mortality	5 (1.1%)	3 (11.1%)	10.9 (2.5–48)	2.5 (0.37–18)

^a^Values obtained including preoperative serum creatinine value, active endocarditis, and CPB duration into a multivariate linear regression; ^b^values obtained including preoperative serum creatinine value, active endocarditis, and CPB duration into a multivariate logistic regression. CI, confidence interval; CPB, cardiopulmonary bypass; HL, hyperlactatemia; ICU, intensive care unit; MV, mechanical ventilation; OR, odds ratio; RR, relative risk.
